# Insights on Host–Parasite Immunomodulation Mediated by Extracellular Vesicles of Cutaneous *Leishmania shawi* and *Leishmania guyanensis*

**DOI:** 10.3390/cells12081101

**Published:** 2023-04-07

**Authors:** Juliana Inês Weber, Armanda Viana Rodrigues, Ana Valério-Bolas, Telmo Nunes, Manuela Carvalheiro, Wilson Antunes, Graça Alexandre-Pires, Isabel Pereira da Fonseca, Gabriela Santos-Gomes

**Affiliations:** 1 Global Health and Tropical Medicine (GHTM), Instituto de Higiene e Medicina Tropical (IHMT), Universidade Nova de Lisboa (UNL), Rua da Junqueira 100, 1349-008 Lisboa, Portugal; 2 Microscopy Center, Faculty of Sciences, University of Lisbon, Campo Grande, 1749-016 Lisboa, Portugal; 3 Research Institute for Medicines, iMed, Faculdade de Farmácia, Universidade de Lisboa, 1649-003 Lisboa, Portugal; 4 Unidade Militar Laboratorial de Defesa Biológica e Química (UMLDBQ), 1849-012 Lisboa, Portugal; 5 CIISA, Centre for Interdisciplinary Research in Animal Health, Faculty of Veterinary Medicine, University of Lisbon, Av. Universidade Técnica, 1300-477 Lisbon, Portugal; 6 Associate Laboratory for Animal and Veterinary Sciences (AL4AnimalS), 2825-466 Setúbal, Portugal

**Keywords:** *Leishmania shawi*, *Leishmania guyanensis*, leishmaniasis, extracellular vesicles, immunomodulation, macrophages

## Abstract

Leishmaniasis is a parasitic disease caused by different species of *Leishmania* and transmitted through the bite of sand flies vector. Macrophages (MΦ), the target cells of *Leishmania* parasites, are phagocytes that play a crucial role in the innate immune microbial defense and are antigen-presenting cells driving the activation of the acquired immune response. Exploring parasite–host communication may be key in restraining parasite dissemination in the host. Extracellular vesicles (EVs) constitute a group of heterogenous cell-derived membranous structures, naturally produced by all cells and with immunomodulatory potential over target cells. This study examined the immunogenic potential of EVs shed by *L. shawi* and *L. guyanensis* in MΦ activation by analyzing the dynamics of major histocompatibility complex (MHC), innate immune receptors, and cytokine generation. *L. shawi* and *L. guyanensis* EVs were incorporated by MΦ and modulated innate immune receptors, indicating that EVs cargo can be recognized by MΦ sensors. Moreover, EVs induced MΦ to generate a mix of pro- and anti-inflammatory cytokines and favored the expression of MHCI molecules, suggesting that EVs antigens can be present to T cells, activating the acquired immune response of the host. Since nano-sized vesicles can be used as vehicles of immune mediators or immunomodulatory drugs, parasitic EVs can be exploited by bioengineering approaches for the development of efficient prophylactic or therapeutic tools for leishmaniasis.

## 1. Introduction

Leishmaniasis is a neglected tropical disease, affecting mainly underdeveloped regions. It is the second disease with the highest mortality rate (only after malaria) and the third in disability-adjusted life years (DALYs), behind malaria and schistosomiasis [1]. This disease becomes even more concerning in a scenario of co-infection, mainly due to the human immunodeficiency virus (HIV) and in transplanted patients, greatly increasing the susceptibility to the disease [2].

Cutaneous manifestations such as localized cutaneous leishmaniasis (LCL), diffuse cutaneous leishmaniasis (DCL), mucocutaneous leishmaniasis (MCL), and post-kala-azar dermal leishmaniasis are usually characterized by inflammatory skin lesions at different levels, which can be disfiguring and very damaging of people’s quality of life [3]. Cutaneous leishmaniasis (CL) becomes even more a concerning disease in patients with HIV co-infection [4,5]. Furthermore, WHO considers a territory endemic if it is registered with at least one autochthonous case and the entire transmission cycle is demonstrated in place, and according to data on the global surveillance of leishmaniasis, by 2020, CL was endemic in 87 countries, with region of the Americas accounting for 19% of the total number of new cases [6]. On the European continent, 47% of the territory was endemic to CL (e.g., France, Bulgaria, Italy, Portugal, Spain, Croatia, Greece, Bosnia-Herzegovina, and Cyprus), and new cases represent about 1% of all cases, including reported cases of HIV/*Leishmania* co-infection [6]. Thus, there is an urgent need to develop new methods of disease control. Since the vertebrate host’s immune system is the first point of contact with the parasite, deepening knowledge about how this interaction occurs is a point of great interest. 

The innate immune response is essential in the defense against *Leishmania* infection. Cells belonging to innate immunity can sense invading organisms through receptors that recognize highly conserved molecular patterns associated with pathogens (PAMPs). These pattern recognition receptors (PRR) include the family of Toll-like receptors (TLR) and nucleotide-binding oligomerization domain (NOD)-like receptors [7].

During the blood meal of the female phlebotomine vector in the vertebrates, *Leishmania* promastigotes are deposited in the dermis of the host, being recognized, and phagocytosed by phagocytes. Upon contact with the pathogen, macrophages (MΦ) are activated and differentiated into professional antigen-presenting cells (APCs), capable of processing and presenting antigens to lymphocytes, establishing the communication between the innate and the adaptive immune response [8]. Promastigotes can activate the MΦ classical pathway (MΦ-M1), producing nitric oxide (NO) and releasing pro-inflammatory cytokines, or activate the MΦ alternative pathway (MΦ-M2), producing urea and polyamines, which are essential for parasite survival in addition to the release of regulatory and anti-inflammatory cytokines. The polarization of macrophages into M1 or M2 phenotypes is dependent on the signals provided by the microenvironment. To avoid being neutralized by active MΦ, parasites must manipulate the host cell to ensure their survival and replication. After pathogen phagocytosis, processed parasite antigens (Ags) are exposed at the APC cell’s surface complexed with molecules of the major histocompatibility complex (MHC), which are responsible for the activation of T lymphocytes [9], driving the adaptive immune response through class I molecules (MHCI) that are recognized by cytotoxic T lymphocytes (CD8^+^ T cells) and class II molecules (MHCII) that are present to T helper lymphocytes (CD4^+^ T cells). 

There is increasing evidence of the immune modulatory effect exerted by *Leishmania* parasites over the host’s immune system. To avoid cytotoxic CD8^+^ T cell activation, the parasite selectively impairs the release of interleukin (IL)-12 and interferon-gamma (IFN-γ). Instead, the parasite directs infected MΦ to produce anti-inflammatory cytokines, such as IL-10, IL-4, and transforming growth factor beta (TGF-β), which are known for their inhibitory action on MΦ functions, promoting the parasite persistence [10,11,12,13]. Taking this collective evidence, the balance between the host and parasite factors that control the activation vs. deactivation of MΦ determines the fate of intracellular parasites. 

An element that has been considered key in host–parasite interactions are the extracellular vesicles (EVs). These nano-sized lipid vesicles, shed by all living cells, generally transport proteins, lipids, and nucleic acids, and have the potential to immunomodulate the target host cells. EVs are classified according to their biogenesis and size. Exosomes originate in the endosomal membrane, constitute multivesicular bodies that fuse with the plasma membrane, and are then shed into the extracellular environment [14]. According to several studies, the diameter of these EVs ranges between 30 and 100 nm. On the other hand, microvesicles are released from the cell membrane, and their size ranges between 100 and 1000 nm [15]. 

Several studies have already evaluated the use of exosomes in the treatment of diseases such as COVID-19 [16,17,18], carcinomas [19], stroke [20], Parkinson’s [21], and Alzheimer’s [22]. In the parasitology field, there are some studies with exosomes from *Plasmodium* [23,24], *Trypanosoma cruzi* [25], *Schistosoma* [26], *Trypanosoma brucei brucei* [27], and *Leishmania* [28]. According to Silverman and collaborators (2008) [29], *Leishmania* EVs can carry virulence factors and modulate MΦ activity to prevent parasite death by inhibiting the secretion of pro-inflammatory cytokines. The main virulence factor present in exosomes is the metalloprotease glycoprotein of 63 kDa (gp63), which can regulate transcription factors of target MΦ, such as NF-κB [30]. However, there are also indications that exosomes also can favor immune protection [31]. Thus, the interest in studies that evaluate the immunomodulation generated by EVs of several pathogens, including *Leishmania,* has been growing, aiming to develop efficient prophylactic and therapeutic strategies [32].

Therefore, the current study aimed to evaluate the effect of EVs shed from two species of *Leishmania* causing CL in the new world into murine MΦ activation. *L. guyanensis* causes different clinical forms varying from LCL to MCL, and *L. shawi* causes a less common CL clinical form. 

## 2. Materials and Methods

### 2.1. Experimental Design

To evaluate the effect of EVs shed by promastigotes of LC-causing *Leishmania* spp. (*L. shawi* and *L. guyanensis*) on murine MΦ cell line, EVs of *Leishmania* axenic promastigotes were isolated from the culture medium and morphologically characterized by Scanning Electron Microscopy (SEM), dynamic light scattering (DLS), and Electrophoretic Light Scattering (ELS). The protein composition of EVs was examined by SDS-PAGE gel and zymography and the interplay of EVs with MΦ was analyzed by multiparametric flow cytometry and fluorescence microscopy. Moreover, to examine the effect of EVs on MΦ immune activation, gene expression of cell sensors and cytokines was evaluated by real-time PCR (RT-qPCR), MHCI and MHCII surface expression was assessed by multiparametric flow cytometry, and NO and urea production was analyzed by colorimetric assays. 

### 2.2. Mouse Macrophage Cell Line

Macrophagic mouse cell line P388D1 (ATCC, Manassas, VA, USA) previously isolated from a mouse lymphoma was maintained in RPMI 1640 culture medium (Biowest^®^, Nuaillé, France) supplemented with 10% inactivated fetal bovine serum (FBS, BioWest^®^), 100 U·mL^−1^ of penicillin and 100 μg·mL^−1^ of streptomycin (Sigma-Aldrich^®^, St. Louis, MO, USA), pH 7.2, at 37 °C in a humid atmosphere with 5% CO_2_. Cells were centrifuged at 300× *g* for 10 min and transferred to a fresh medium supplemented with 10% exosome-depleted inactivated FBS (exo-free FBS, Exosome-Depleted Fetal Bovine Serum, Gibco, Waltham, MA, USA) to be used in the following assays.

### 2.3. Leishmania Cultures

*L. shawi* (MHOM/BR/96/M15789) and *L. guyanensis* (M19663) (SNGPGCTA—certificate ref. A095CE9) promastigotes were maintained in Schneider’s *Drosophila* medium (Biowest^®^), supplemented with 10% heat-inactivated FBS, 100 U·mL^−1^ of penicillin, and 100 μg·mL^−1^ of streptomycin and were incubated at 26 °C. Parasites in the logarithmic growth phase were centrifuged at 1800× *g* for 10 min and further used to isolate EVs and also to infect MΦ. 

### 2.4. Isolation of EVs Shed by Leishmania Promastigotes

Viable promastigotes of *L. shawi* and *L. guyanensis* kept in Schneider’s medium were centrifuged at 1800× *g.* A fresh medium supplemented with exo-free FBS was added, and parasites were incubated at 26 °C for 24 h. Cultures were again centrifuged at 1800× *g* for 10 min, and the pellet was transferred to the fresh medium and incubated at 26 °C for 72 h. After this period, the cultures were centrifuged at 1800× *g* for 10 min to remove the parasites, and supernatants were further centrifugated at 2000× *g* for 30 min to remove cellular debris. The supernatant was collected, and the exosome isolation reagent (Invitrogen, Carlsbad, CA, USA) was added at a ratio of 1:2, according to the manufacturer’s instructions, and the supernatant was incubated for 24 h at 4 °C. After incubation, the EV solution was centrifuged at 10,000× *g* for 1 h at 4 °C. Pellets (rich in EVs) were resuspended in 1× phosphate-buffered saline (PBS) and used immediately or stored at −80 °C for further assays. In parallel, sterile Schneider’s medium supplemented with 10% exo-free FBS followed the same protocol of EV isolation, and the obtained solution was used as a negative control of the EV isolation method (ImC). The proteins in the final EV solution were quantified in the NanoDrop 1000^®^ spectrophotometer (Thermo Scientific, Waltham, MA, USA).

### 2.5. Production of L. shawi and L. guyanensis Soluble Antigens 

*L. shawi* and *L. guyanensis* promastigotes of the logarithmic growth phase were centrifuged at 1800× *g* for 10 min, and the obtained pellet was washed twice with 1× PBS. The supernatants were discarded, and the pellet was resuspended in 200 μL of 1× PBS. The parasites were subjected to three cycles of freezing and thawing followed by shaking to promote cell lysis and the release of soluble proteins. After these cycles, the lysed parasites were centrifuged at 1800× *g* for 10 min, and the total soluble protein of the supernatants was quantified in the NanoDrop. *L. shawi* and *L. guyanensis* soluble antigens (Ags) were stored at −80 °C until further use.

### 2.6. Characterization of Extracellular Vesicles

The topography analysis of the EVs was performed using scan electron microscopy (SEM). For this analysis, EVs isolated from *L. shawi* and *L. guyanensis* promastigotes were used, as well as viable promastigotes of both species. 

For the promastigotes, round glass coverslips were immersed into poly-D-Lysine (Sigma-Aldrich^®^) overnight to increase adherence and later placed in a 24-well plate. Then, parasites were left to adhere to the coverslips, followed by a fixation step with PBS 4% paraformaldehyde (Merck, Rahway, NJ, USA) for 30 min at 4 °C. For EVs, coverslips were rinsed three times with distillate water, treated with 0.5% osmium tetroxide (Sigma-Aldrich^®^), and washed again. A fixative solution of 1% tannic acid (Sigma-Aldrich^®^) was added for 30 min. EVs were fixed to coverslips with 2.5% glutaraldehyde, 0.1 M sodium cacodylate buffer, and pH 7.4 for 2 h at 4 °C, and then coverslips were washed. Afterward, both parasites- and EV-coverslips were washed and dehydrated by sequential addition of 30%, 50%, 70%, 80%, and 90% ethanol for 5 min each. Coverslips were immersed in 100% ethanol and then treated with hexamethyldisilane solvent (Sigma-Aldrich^®^), coated with gold-palladium, and mounted on stubs to be observed under an ultra-high resolution scanning electron microscope (Hitachi SU8010, Hitachi High-Technologies Corporation, Tokyo, Japan). Acquired images were analyzed using ImageJ software to estimate the vesicle diameter.

The diameter of purified EVs in 1× PBS pH 7.5 was analyzed by dynamic light scattering (DLS) in Malvern ZetaSizer equipment (Nano-S, Malvern Instruments, Malvern, UK), at a constant temperature of 25 °C and with a detector placed at 90°. EV zeta potential (ζ), which is related to membrane charge and is an important indicator of the stability of colloidal dispersion, was evaluated using electrophoretic light scattering (ELS) at pH 7.5 in a Malvern ZetaSizer equipment (Nano-Z, Malvern Instruments). ImC was also analyzed for the diameter and zeta potential of its constituents.

### 2.7. EV Proteins 

Protein characterization of EVs was performed by acrylamide gel electrophoresis (10%) with sodium dodecyl sulfate (10% SDS-PAGE) and zymography (with 0.41% gelatin). *L. shawi* and *L. guyanensis* EVs (50 μg of protein) were added to the gel, along with a 4× loading buffer (0.25 M Tris, 8% SDS, 10% glycerol, 2% bromophenol blue) supplemented with 1:10 of β-mercaptoethanol. ImC was also used to disclose protein components that did not correspond to parasite EVs. For the zymography assay, *L. shawi* and *L. guyanensis* EVs (50 µg of protein) were added to the gel with a 4× loading buffer free of β-mercaptoethanol. 

After the end of the run, the SDS-PAGE was stained with Coomassie^®^ Brilliant Blue G 250 (Sigma-Aldrich^®^) and destained with a solution of 10% acetic acid and 40% methanol to visualize the protein bands. For the zymography assay, the gel was incubated with Triton-X100 for 1 h and then incubated for 18 h with 0.5M Tris-HCl buffer (pH 7.5), 0.2 M NaCl, 0.005 M CaCl_2,_ and 0.02% Brij35. Afterward, the gel was stained and destained following the same steps of SDS-PAGE. 

The molecular mass of the bands found in ImC and EV samples was determined by comparison with the 10–250 kDa molecular weight (MW) marker (Precision Plus Protein Dual Color Standards, Bio-Rad, Hercules, CA, USA) using the GelAnalyzer 19.1 software (www.gelanalyzer.com).

### 2.8. Interplay of Extracellular Vesicles and Macrophages

To follow the interplay of parasite EVs with MΦ, the lipophilic fluorescent cationic dye DiR’. DilC_18_(7) (1,1′-Dioctadecyl-3,3,3′,3′-Tetramethylindotricarbocyanine Iodide, Thermo Fisher^®^, Waltham, MA, USA), which incorporates into lipid membranes increasing fluorescence, was used to EV stains.

EVs were incubated with DilC_18_ for 2 h at 26 °C and then were passed through columns (Exosome Spin Columns, MW3000, Invitrogen, USA) and centrifuged at 750× *g* for 3 min to remove the unincorporated dye. In parallel, 1× PBS was incubated with DilC_18_ and passed through the column to be used as a negative staining control and to assess the capacity of the column to retain non-bonded DilC_18_. On the other hand, MΦ were directly incubated with the dye to be used as a positive control. Stained EVs and the negative control were incubated with MΦ for 4 h, 24 h, and 48 h at 37 °C in a humid atmosphere with 5% CO_2_. At each time point, negative and positive as well as EVs-incubated MΦ were washed with 1× PBS, and samples were then analyzed by multiparametric flow cytometry and fluorescence microscopy. Samples were acquired by a flow cytometer analyzer (CytoFlex, Beckman Coulter, Brea, CA, USA), and the proportion of positive cells, as well as the mean fluorescence intensity (MFI), were evaluated.

For microscopy examination, cells were fixed with 2% paraformaldehyde for 30 min at 4 °C, and MΦ nuclei were stained with DAPI (Fluroshield^TM^ with DAPI, Sigma-Aldrich^®^). The slides were observed under a fluorescence microscope (Eclipse 80i Intensilight C-HGFI with NIS-Elements software, Nikon, Japan), and images were acquired. 

### 2.9. Effect of Extracellular Vesicles on Macrophages Activity

To evaluate the effect of parasite EVs on MΦ activity, cells (1 × 10^6^ cells·mL^−1^) were separately incubated at 37 °C in a humid atmosphere with 5% CO_2_, for 4 h, 24 h, and 48 h, with (i) viable promastigotes of *L. shawi* or *L. guyanensis* (ratio cell: promastigote 1:3), (ii) 40 μg·mL^−1^ of *L. shawi* or *L. guyanensis* soluble Ag, and (iii) *L. shawi* (LsEVS) or *L. guyanensis* (LgEVs) EVs at concentrations of 5, 10, 20, and 45 μg·mL^−1^. In parallel, (iv) resting MΦ and (v) MΦ stimulated with Phorbol-12-myristate-13-acetate (PMA, Sigma-Aldrich^®^ ) at 0.2 µg·mL^−1^ were also evaluated.

After incubation, MΦ were centrifuged at 300× *g*, the supernatants were collected and stored at −20 °C for further quantification of NO and urea, and cells were used for determination of MΦ viability, immunophenotyping, and real-time PCR. 

### 2.10. Macrophage Viability after Exposure to EVs

To assess the viability of MΦ that were incubated with promastigotes or EVs, resazurin (7-hydroxy-3H-phenoxazin-3-one-10-oxide) metabolization assay (Sigma-Aldrich^®^) was used. Resazurin is a low-fluorescent blue compound that is reduced to resorufin by metabolically active cells, resulting in highly fluorescent pink staining [33]. 

MΦ were added to 96-well plates at a concentration of 1 × 10^6^ cells/mL, along with different concentrations of EVs (5, 10, 20, and 45 µg·mL^−1^), 40 µg·mL^−1^ soluble Ag, and promastigotes (1:3), and were incubated at 37 °C with 5% CO_2_ for 24 h, 48 h, and 72 h. Three controls were used: resting MΦ (negative control, NC), MΦ stimulated by 0.2 µg·mL^−1^ phorbol myristate acetate (PMA), and cell death control (DC) where 2% paraformaldehyde was added to MΦ. Paraformaldehyde inactivates cell metabolism, and these cells are considered non-viable cells. Then, resazurin solution in 1× PBS (0.067 µg·mL^−1^) was added to all wells and incubated for 1 h at 37 °C. After incubation, absorbances were read at 595 nm, after excitation at 535 nm, in a TRIADTM 1065 fluorimeter (DYNEX Technologies, Chantilly, VA, USA). 

### 2.11. Urea and Nitric Oxide Production

To evaluate the final products that result from the activation of L-arginine pathways, MΦ supernatants from all experimental conditions (as described in 2.9) were centrifuged to remove cell debris and used to quantify urea and NO production using the Urea Assay Kit (BioChain^®^, Newark, CA, USA) and Nitrate/Nitrite colorimetric assay kit (Cayman Chemical, Ann Arbor, MI, USA), respectively, according to the manufacturer’s instructions. The chromogenic reagent present in the urea kit reacts specifically with urea, developing a colorimetric complex that can be analyzed by spectroscopy at a wavelength of 430 nm (TRIADTM 1065 fluorimeter (DYNEX Technologies), a color intensity that is directly proportional to the concentration of urea in the sample. The nitrate/nitrite concentration was determined using a two-step process: first, it converts nitrate to nitrite using the nitrate reductase, and in the second step utilizes the Griess Reagent to convert the nitrite into the azo compound with the color purple that can be photometrically measured for absorbance at 540 nm. The final results were normalized to an RPMI-supplemented medium and presented as fold change to resting MΦ (non-stimulated cells). 

### 2.12. MHCI and MHCII Expression on the Macrophage Surface

Expression of MHCI and MHCII molecules on the surface of MΦ exposed to parasites and stimulated by PMA, Ag, and EVs and in resting MΦ was analyzed by multiparametric flow cytometry. After 24 h, 48 h, and 72 h of incubation, cells were harvested and washed three times with 1× PBS and mouse-monoclonal MHCI (FICT, Thermo Fisher, clone 34-1-2S), and MHCII (PE, Thermo Fisher, M5/114.15.2) antibodies diluted in 1× PBS 2% albumin (*w*/*v*) were added (2:100 and 0.1:100, respectively). Cells were acquired by a flow cytometer (CytoFlex), and MFI (median fluorescence intensity) values were analyzed and presented as fold change to resting MΦs (non-stimulated cells).

### 2.13. Gene Expression of Cytokines and Cell Sensors

To evaluate the relative gene expression of Toll-like and NOD-like innate immune receptors in MΦ exposed to parasites and stimulated by EVs, Ag, and PMA as well as the generation of pro-inflammatory and anti-inflammatory interleukin *(IL-)1β, IL-4, IL-10, IL-12p40,* and *tumor necrosis factor (TNF)-α*, the total RNA was extracted using the RNA extraction kit (NZY Total RNA Isolation Kit, NzyTech, Lisbon, Portugal) following the manufacturer’s instructions. The quantity and purity of isolated RNA were evaluated in the NanoDrop. cDNA synthesis was performed using NZY first-strand cDNA synthesis kit (NzyTech), followed by real-time semi-quantitative RT-PCR gene expression analysis using primers specific for mouse MΦ (Supplementary Table S1). Primer efficiency was between 90 and 110% for all primers used. For real-time semi-quantitative PCR, it was performed in a mix of 10 µL of SsoAdvanced Universal SYBR^®^ Green Supermix (Bio-Rad, Hercules, CA, USA), 0.15 µL of each primer (forward and reverse), 2 µL of sample cDNA, and 7.7 µL of ultra-pure water. Samples were then amplified in the BioRad thermocycler (CFX Connect BioRad). Gene amplification conditions included 39 cycles of denaturation (95 °C for 5 min, 95 °C for 30 s) and annealing for 30 s. Finally, the extension was performed at 50 °C for 15 min. The housekeeping gene *HPRT* was used to perform a baseline of gene expression in each analyzed sample (ΔCt). Resting cells (non-stimulated macrophages) were collected at each time point and considered as the negative control and used to perform the relative quantification at each time point (∆∆Ct). The results of the relative analysis were obtained through the formula 2^−∆∆Ct^ [34].

### 2.14. Data Analysis

Three independent experiences with a minimum of triplicates per experimental condition were performed. After verifying the normality of the sample by the Shapiro–Wilk test, the parametric Student’s *t*-test was used to compare the means between the two groups and analyze the differences between the experimental conditions and the controls of each method. The unidirectional ANOVA test was used to compare the mean among samples in groups in the following situations: (i) to analyze the effect of the time (24 h, 48 h, and 72 h) on the same experimental conditions and (ii) to analyze the statistical significance of the crescent EV concentrations for a defined *Leishmania* species. A significance level of 5% (*p* < 0.05) was used as indicative of statistical significance. Data analysis was performed using the GraphPad Prism 9 software (San Diego, CA, USA).

## 3. Results

### 3.1. Extracellular Vesicles Shed by L. shawi and L. guyanensis Promastigotes Are Compatible with Exosomes and Microvesicles

**Figure 1:**
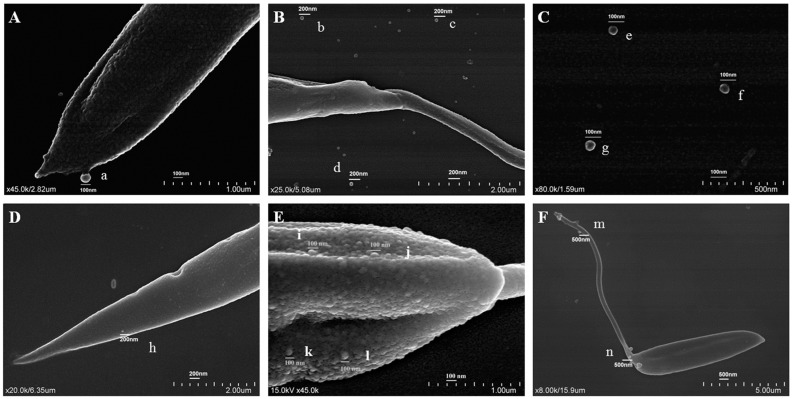
Topography of promastigotes and EVs of *L. shawi* and *L. guyanensis***.** Scanning electron microscopy of cultured *L. shawi* (**A–C**) and *L. guyanensis* (**D–F**) promastigotes exhibiting protrusions compatible with EVs biogenesis (**A,D–F**). Free EVs (**B,C**) with less than 100 nm can also be observed.

**Figure 2:**
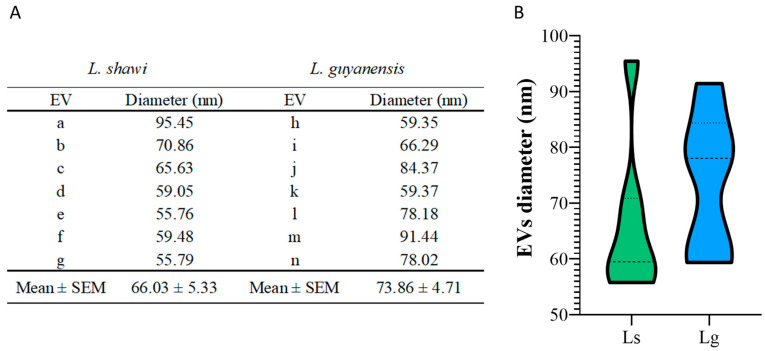
Diameter of EVs shed by *L. shawi* and *L. guyanensis* promastigotes. The diameter of EVs observed in Figure 1 were analyzed and are shown in table (**A**). a to g are measurement of EVs from *L. shawi* and h to n are values obtained for *L. guyanensis* EVs with Mean and standard error of the mean (SEM). EVs diameter (nm) distribution is shown by the violin plot (**B**).

Topographic observation of promastigotes showed EVs budding throughout the body of the parasite (Figure 1A,B,D–F). EVs isolated from the culture medium of *L. shawi* and *L. guyanensis* promastigotes appear mostly spherical, with a smooth membrane (Figure 1C) and exhibited diameters ranging between 95.45 and 55.76 nm, which is consistent with the size described for exosomes (Figure 2). 

**Figure 3:**
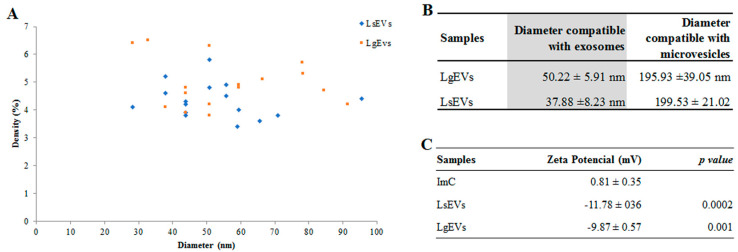
Diameter, density, and zeta potential of *L. guyanensis* and *L. shawi* EVs. The density and diameter of EVs shed by *L. shawi* (blue) and *L. guyanensis* (orange) are represented in a scatter plot (**A**). The mean and the standard deviation of six independent EV isolations and three readings per sample are indicated in Tables (**B**,**C**). Size of *L. shawi* and *L. guyanensis* EVs (**B**) and the zeta potential (**C**) were analyzed by ZetaSizer. ImC was applied as a control of the EV extraction method. Parametric Student’s *t*-test (*p* ≤ 0.05) was used to compare the zeta potential of EVs and ImC.

The analysis of EVs by DLS confirmed the presence of nanoparticles (vesicles) with a variable size dispersion compatible with the presence of exosomes (30–100 nm) and microvesicles (100–2000 nm). The EV profile observed was similar between the two species of *Leishmania,* with only small, non-statistically significant variations between the size and density noticed (Figure 3A). DLS analysis of *L. shawi* and *L. guyanensis* EV samples showed the presence of vesicles with a diameter compatible with exosomes (LsEVs—37.88 ± 8.23 nm and LgEVs—50.22 ± 5.91 nm) and also the presence of vesicles with a diameter compatible with microvesicles (LsEVs—199.53 ± 21.02 and LgEVs—195.93 ± 39.05 nm) (Figure 3B and Supplementary Figure S1A,B). 

The analysis of the zeta potential (ζ,) defined as the voltage at the edge of the diffuse layer where it meets the surrounding liquid and, therefore, indicative of the presence of intact lipid membranes in suspension, revealed important differences between the EVs extracted from promastigotes and the negative control (ImC) (Figure 3C). The zeta potential of EVs isolated from *L. shawi* and *L. guyanensis* showed negative values, with an average of −11.78 ± 0.36 mV and−9.87 ± 0.57 mV, respectively. When compared to ImC (control), there were statistically significant differences, pointing out to the successful isolation of intact *Leishmania*-derived EVs (LsEVs *p* = 0.0002 and LgEVs *p* = 0.001). Since most cellular membranes are negatively charged, the zeta potential of a nanoparticle can influence its tendency to interact and permeate other cell membranes. However, nanoparticles with a zeta potential between−10 and +10 mV are considered approximately neutral [32], suggesting that *Leishmania* EVs can interact with other cell membranes in a non-disruptive way. In addition, the range of zeta potential obtained for *Leishmania* EVs is indicative of their ability to flocculate, generating EV aggregates that may promote their interaction with cell membranes. 

### 3.2. L. shawi and L. guyanensis EVs Carry Active Proteinases 

**Figure 4:**
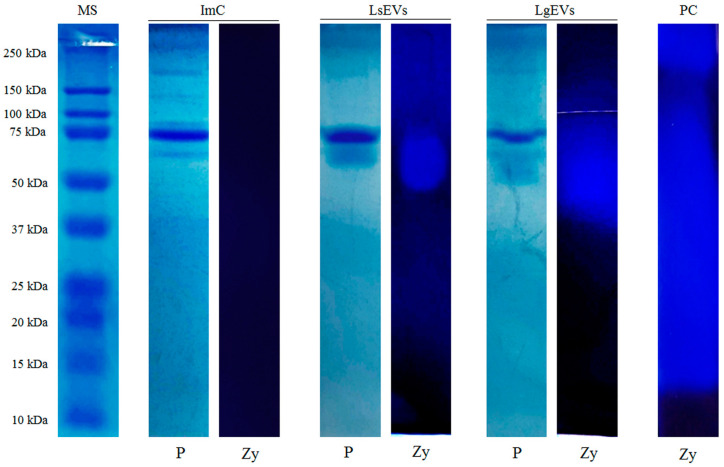
Protein constitution and proteolytic activity of EVs from *L. shawi* and *L. guyanensis.* EVs were evaluated by SDS-PAGE (P) and by zymography (Zy), and images of the strips were acquired. MS: molecular weight size marker. ImC: isolation method control (Supplemented Schneider’s medium extracted with exosome isolation reagent); LsEVs—EVs from *L. shawi* promastigotes; LgEVs: EVs from *L. guyanensis* promastigotes; PC: positive control of proteolytic activity (1× trypsin).

The evaluation of the EV protein profiles from both *L. shawi* and *L. guyanensis* showed the presence of four protein fractions with molecular masses of approximately 50 kDa, 63 kDa, 70 kDa, and 80 kDa (Figure 4). These protein fractions appeared to be exclusively present in EV samples since they were not identified in the ImC. However, the SDS-PAGE assay showed the presence of six bands associated with the ImC protein profile with molecular mass ranging between 56 and 267 kDa (Figure 4, ImC strip). These protein fractions most likely correspond to proteins present in Schneider’s medium due to its supplementation with FBS (exo-free), such as bovine albumin (with described molecular weight of 66.5 kDa). These bands also appeared to be present in EVs samples isolated from promastigotes growing in supplemented Schneider’s medium, but when compared to the other bands, these protein fractions were not as evident, suggesting some catabolic degradation by the parasites in the culture. Moreover, the zymogram assay showed proteolytic activity associated with proteins ranging from 50 kDa to 80 kDa for both *Leishmania* isolated EVs and no proteolytic activity in ImC fractions. Overall, the methodology used was able to isolate EVs with intact membranes and active proteolytic activity, although with some protein contaminants from the medium. Interestingly, the molecular mass, together with the proteolytic activity, are suggestive of the presence of glycoprotein 63 kDa (gp63), which is considered an important virulence factor of *Leishmania* parasites and has also been identified in EVs of other species of *Leishmania*.

### 3.3. EVs Are Rapidly Taken up by Murine Macrophages

**Figure 5:**
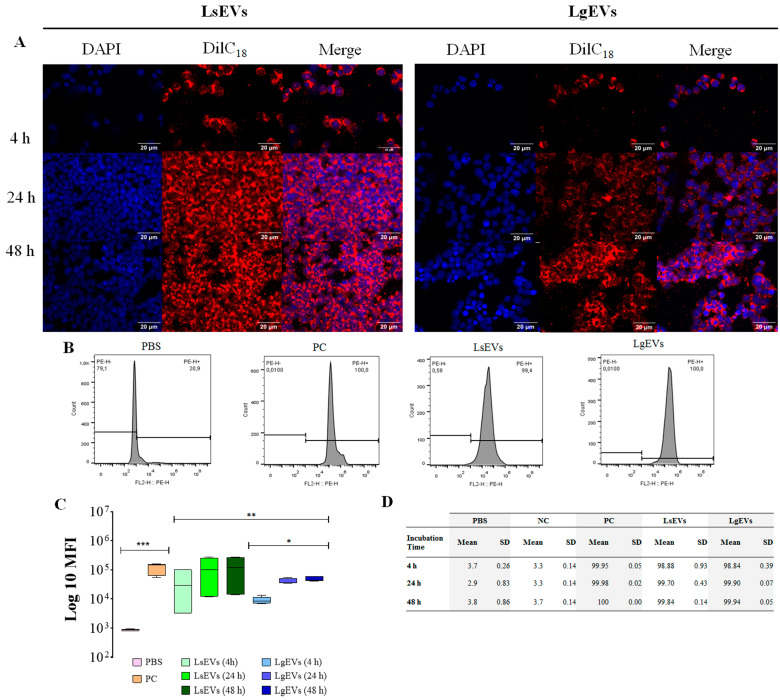
Macrophage incorporation of *L. shawi* and *L. guyanensis* EVs. EVs of *L. shawi* (LsEVs) and *L. guyanensis* (LgEVs) were isolated, stained with DilC_18_, purified with exosome spin columns (MW3000, Invitrogen, USA), and incubated with MΦ for 4 h, 24 h, and 48 h. Fluorescence microscope images of MΦ stained with DAPI (blue) and incubated with DilC_18_ (red) labeled EVs were acquired (600× magnification) (**A**). DilC_18_ positive cells were analyzed by multiparametric flow cytometry, and median fluorescence intensity (MFI) (**B**,**C**) and the frequency of positive stained-MΦ were registered (**D**). Student’s parametric *t*-test was used for statistical analysis. * (*p* < 0.05), ** (*p* < 0.01) and *** (*p* < 0.0001) indicate statistical significance. As the negative control (NC) of the assay, 1× PBS was incubated with DilC_18_, purified through the column, and added to resting (non-stimulated) MΦ. For positive control (PC), DilC_18_ was directly used to stain the membranes of resting MΦ.

To analyze how *Leishmania* EVs interacted with phagocytic cells, DilC_18_-stained EVs were incubated with murine P388D1 macrophages. The observation of cells by fluorescence microscopy reveals that after 4 h of incubation *L. guyanensis* and *L. shawi* EVs were incorporated by MΦ. EV incorporation by MΦ was visible through the accumulation of stained vesicles bonded to the cells (Figure 5A and in more detail in Supplementary Figure S2) that increased with incubation time, suggesting the probable fusion of EVs with MΦ membranes, resulting in fluorescence increase. 

The uptake of DilC_18_ stained *L. guyanensis* and *L. shawi* EVs by MΦ was also followed by flow cytometry analysis. After 4 h of incubation, MΦ showed 98.84% and 99.05% of LsEVs and LgEVs fluorescent cells, respectively. These values were maintained during the 48 h of observation. In contrast, unstained MΦ and ImC (negative controls) evidence residual fluorescence (Figure 5B). 

MΦ incubated with stained LsEVs and LgEVs for 4 h showed a higher MFI increase when compared with unstained MΦ (*p* < 0.0001), reaching maximum values at 24 h and maintained at 48 h. Interestingly, LsEVs incubated MΦ showed higher MFI when compared with MΦ incubated with LgEVs (*p* = 0.0009) (Figure 5C). 

Taken together, these results indicated that MΦ fast incorporates EVs and that the density of LsEVs incorporation is more intense in comparison with LgEV. 

### 3.4. EVs Do Not Affect Macrophages’ Viability

After protein analyses of *L. guyanensis* and *L. shawi* EVs and before evaluating the effect of these nano-sized vesicles on the immunological activity of MΦ, the potential effect of EVs, promastigotes, and parasite Ags on MΦ viability were assessed. Resting MΦ was considered to represent 100% viability.

**Figure 6:**
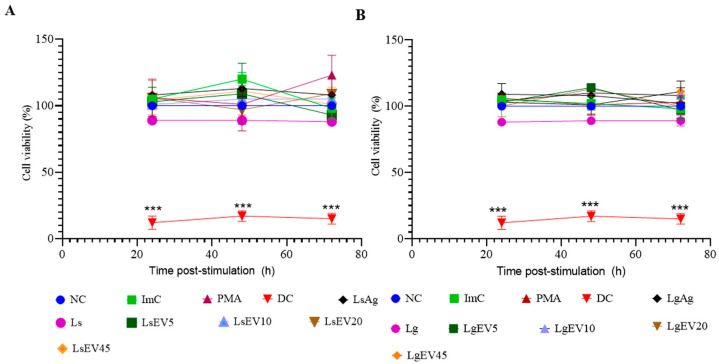
Effect of *L. shawi* and *L. guyanensis* EVs on MΦ viability. The viability of MΦ incubated for 24 h, 48 h, and 72 h with 5, 10, 20, and 45 µg·mL^−1^ of EVs from *L. shawi* (**A**) and *L. guyanensis* (**B**), parasite antigens, and promastigotes was analyzed by resazurin reduction. NC: negative control (resting MΦ); ImC: control of EV extraction method; PMA: positive control of inflammation (PMA 0.2 µg·mL^−1^); DC: MΦ death control (2% paraformaldehyde); LsAg: *L. shawi* antigen; Ls: *L. shawi* promastigotes; LsEV5: *L. shawi* EVs at 5 µg·mL^−1^, LsEV10: *L. shawi* EVs at 10 µg·mL^−1^; LsEV20: *L. shawi* EVs at 20 µg·mL^−1^; LsEV45: *L. shawi* EVs at 45 µg·mL^−1^; LgAg: *L. guyanensis* antigen; Lg: *L. guyanensis* promastigotes; LgEV5: *L. guyanensis* EVs at 5 µg·mL^−1^; LgEV10: *L. guyanensis* EVs at 10 µg·mL^−1^; LgEV20: *L. guyanensis* EVs at 20 µg·mL^−1^; LgEV45: *L. guyanensis* EVs at 45 µg·mL^−1^. The mean and standard deviation of three independent assays performed in triplicate are represented by dot plots with connecting lines. Student’s parametric *t*-test was used for statistical analysis. *** indicates significant differences (*p* ≤ 0.001) when compared to DC.

When compared to the resting MΦ, it was observed that during 72 h of exposure, EVs, as well as parasite Ag, did not alter the MΦ viability (Figure 6). The presence of promastigotes, on the other hand, could lead to a slight reduction in the MΦ viability, although not statistically significant. On the other hand, death control showed a significant difference to all accessed experimental conditions (*p* < 0.001). Remarkably, *Leishmania* EVs did not alter MΦ viability significantly, although they interacted directly with MΦ cell membrane as previously observed (see Section 3.3). 

### 3.5. Leishmania EVs Modulate Macrophages to Generate TLR2, TLR9, NOD1, and NOD2

MΦ innate immune sensors can recognize parasite antigens, signaling downstream pathways that can lead to MΦ immune activation, resulting in the synthesis of immune mediators. Therefore, the gene expressions of cell membrane *TLR2* and *TLR4*, of endocytic membrane *TLR9* and cytoplasmatic *NOD1* and *NOD2* were evaluated in MΦ exposed to EVs and parasites in comparison with PMA stimulated MΦ (inflammatory stimulation). 

**Figure 7:**
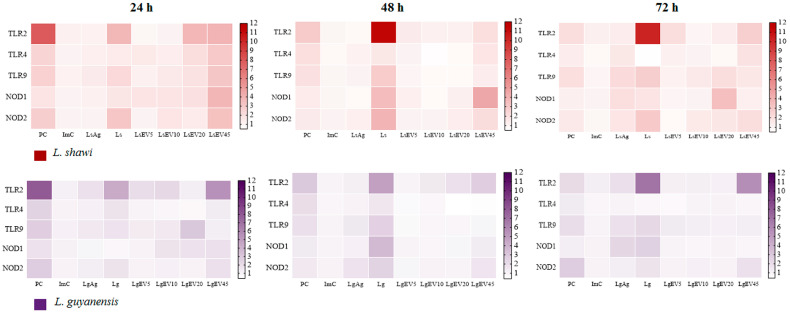
Effect of *L. shawi* and *L. guyanensis* EVs on *TLR2, TLR4, TLR9, NOD1,* and *NOD2* gene expression. MΦ incubated for 24 h, 48 h, and 72 h with 5 (LsEV5; LgEV5), 10 (LsEV10; LgEV10), 20 (LsEV20; LgEV20), and 45 µg·mL^−1^ (LsEV45; LgEV45) of EVs from *L. shawi* (red) and *L. guyanensis* (purple), parasite antigens (LsAg and LgAg), and *L. shawi* (Ls) and *L. guyanensis* (Lg) promastigotes were analyzed by RT-qPCR. Results normalized to resting MΦ of three independent assays performed in triplicate are represented by heat maps. PC—positive control (MΦ incubated with PMA); ImC—MΦ incubated with negative control of EV isolation method.

After 24 h of incubation (Figure 7), PMA stimulated MΦ showed a *TLR2, TLR4, TLR9*, *NOD1*, and *NOD2* fold increase, evidencing that MΦ were able to express these innate immune pattern recognition receptors (PRRs). In contrast, parasite Ag induced a low gene expression of these sensors. However, after 72 h of incubation, *L. shawi* Ag caused an increase in *TLR9* gene expression (*p* = 0.0087). 

Regarding the innate immune receptors, the greatest upregulation of *TLR2, TLR9*, *NOD1*, and *NOD2* genes was observed in MΦ exposed to *L. shawi* promastigotes or LsEVs. Interestingly, both parasites promoted increasing levels of intracellular *TLR9* (*L. shawi*: p_24h_ = 0.0037, p_48h_ = 0.0128, p_72h_ = 0.0099; *L. guyanensis*: p_24h_ = 0.0215, p_48h_ = 0.0448, p_72h_ = 0.0058) and cytoplasmic *NOD1* (*L. shawi*: p_24h_ = 0.0065, p_48h_ = 0.0067, p_72h_ = 0.0750; *L. guyanensis*: p_48h_ = 0.0040, p_72h_ = 0.0362) at all time points, suggesting parasite internalization by MΦ.

An increase in *TLR2* gene expression was detected in MΦ stimulated with 45 µg·mL^−1^ of EVs at all time points (*L. shawi*: p_24h_ = 0.0041, p_48h_ < 0.0001, p_72h_ = 0.0002; *L. guyanensis*: p_24h_ = 0.0004, p_48h_= 0.0027, p_72h_ = 0.0007). In contrast, low generation of *TLR4* and *NOD2* was observed in EV-stimulated MΦ during the 72 h of the study.

MΦ stimulated with LsEV45 exhibited an early increase in intracellular PRRs *TLR9* and *NOD1* (*p*= 0.0385) gene expression, followed by a decrease after 48 h of stimulation. The highest concentration of LgEVs promoted the upregulation of cytoplasmic *NOD2* (*p* = 0.0309) gene expression. Thus, these results indicated that parasite EVs could be recognized by macrophage PRRs. 

However, differences in the MΦ’s profiles were observed among these two *Leishmania* species. *L. shawi* promastigotes and LsEVs were more efficient in modulating the cell membrane *TLR2* (*p* = 0.0002), the endocytic *TLR9* (*p*= 0.0152), and the cytoplasmatic *NOD1* (*p* < 0.0001) and *NOD2* (*p* < 0.001) when compared with *L. guyanensis*. Overall, at 24 h of incubation, the higher concentration of LsEVs exerted a more pronounced effect on MΦ innate immune receptors. 

### 3.6. Parasite EVs Modulate MΦ’s to Generate Pro- and Anti-Inflammatory Cytokines 

MΦ are the *Leishmania* host cell and also make part of the first line of defense against these parasites. Upon stimulation, MΦ can synthesize pro- and anti-inflammatory cytokines that direct the immune activation of other cells. Thus, the effect of EVs shed by *L. shawi* and *L. guyanensis* parasites on MΦ gene expression of proinflammatory cytokines (*IL-1β, IL-12*, and *TNF*-α) and anti-inflammatory cytokines (*IL-4* and *IL-10*) was examined. 

**Figure 8:**
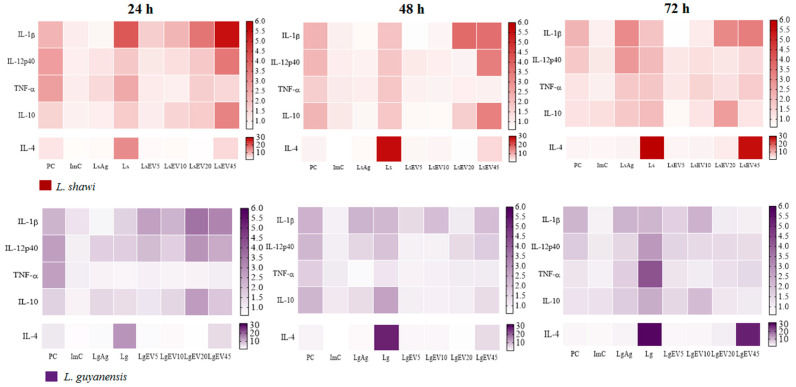
Effect of *L. shawi* and *L. guyanensis* EVs on cytokines gene expression. MΦ incubated for 24 h, 48 h, and 72 h with 5 (LsEV5; LgEV5), 10 (LsEV10; LgEV10), 20 (LsEV20; LgEV20), and 45 µg·mL^−1^ (LsEV45; LgEV45) of EVs from *L. shawi* (red) and *L. guyanensis* (purple), parasite antigens (LsAg and LgAg), and *L. shawi* (Ls) and *L. guyanensis* (Lg) promastigotes were analyzed by RT-qPCR. Results normalized to resting MΦ are represented by heat maps of three independent assays performed in triplicate. PC—positive control (MΦ incubated with PMA); ImC—MΦ incubated with negative control of EV isolation method.

During 24 h to 48 h of stimulation, PMA induced a significant increase of proinflammatory cytokines and *IL-4*, indicating that MΦ were able to generate cytokines (*p* < 0.0001) (Figure 8).

After 72 h of stimulation, *L. shawi* Ag promoted a significant increase in *IL-1β* (*p* = 0.0049) and *IL-12p40* (*p* = 0.0014), and *L. guyanensis* Ag caused an increase in *IL-1β* at 48 h (*p* = 0.0334) and 72 h of stimulation (*p* = 0.0098). At 24 h of incubation, *L. shawi* parasites promoted the upregulation of *IL-1β* (*p* = 0.0063) and *TNF-α* (*p* = 0.0070) gene expression. Increased *IL-1β* gene expression was also observed after 72 h (*p* = 0.0174). However, *L. guyanensis* promastigotes needed 72 h to induce the upregulation of *IL-1β* (*p* = 0.0082), *IL-12p40* (*p* = 0.0016), and *TNF-α* (*p* = 0.0081). *IL-4* gene expression was stimulated at all time points by both parasite species (*L. shawi*: p_24h_ = 0.0001, p_48h_ = 0.0051, p_72h_ = 0.0398; *L. guyanensis*: p_24h_ = 0.0236, p_48h_ = 0.0474, p_72h_ = 0.0086). After 48 h (*p* = 0.0255) and 72 h (*p* = 0.0328) of exposure to *L. guyanensis* parasites, MΦ evidenced the upregulation of *IL-10* gene expression. 

Although presenting fluctuations related to stimulation time and concentration, *L. shawi* and *L. guyanensis* EVs induced in MΦ an increase in *IL-1β* generation. The higher concentrations of LsEVs (20 µg·mL^−1:^ p_24h_ = 0.0054, p_48h_ = 0.0106, p_72h_ = 0.0290, 45 µg·mL^−1^: p_24h_ = 0.0387, p_48h_ = 0.0207, p_72h_ = 0.0335) induced *IL-1β* upregulation at all time points. MΦ stimulated with LgEVs also exhibited the upregulation of IL-1β after 24 h of stimulation (*p*_LgEV20_ = 0.0096 and p_LgEV45_ = 0.0400), and the lowest concentration of LgEVs was also able to induce a sustained increase in *IL-1β* gene expression (p_24h_ = 0.0447; p_48h_ = 0.0443; p_72h_ = 0.0334). 

Increased gene expression of *IL-12p40*, a subunit of IL-12 and IL-23 proinflammatory cytokines, was induced early by EVs. After 24 h of stimulation, the two highest concentrations of LgEVs upregulated *IL-12p40*, followed by a decrease (p_LgEV20_ = 0.0153, p_LgEV45_ = 0.0273). MΦ stimulated with 20 (*p* = 0.0127) and 45 µg·mL^−1^ (*p* = 0.0086) of LsEVs also showed an early increase in *IL-12p40* gene expression. However, the highest concentration of LsEVs promoted the upregulation of this cytokine for 48 h (*p* = 0.0057), followed by a significant decrease (p_72h_ < 0.0001). In contrast, downregulation of *TNF-α* was found in MΦ exposed to EVs. Moreover, EVs highly induced *IL-4* upregulation, which can interfere with the inflammatory immune response. MΦ stimulated with the higher concentration of LsEVs and LgEVs showed the increase of *IL-4* gene expression at all time points (*L. shawi*: p_24h_ = 0.0287, p_48h_ = 0.0179, p_72h_ = 0.0040; *L. guyanensis*: p_24h_ = 0.0497, p_48h_ = 0.0424, p_72h_ = 0.0040).

Parasite EVs also promote MΦ to upregulate *IL-10*, a regulatory cytokine that contributes to the balance of immune response. During the first 48 h of stimulation, the highest concentration of LgEVs induced MΦ to a transitory *IL-10* upregulation (*p* = 0.0098). Furthermore, at 72 h of stimulation, 20 µg·mL^−1^ of LsEVs led to a significant increase of *IL-10* (*p* = 0.0175). The two highest concentrations of LgEVs also caused an early transitory increase in *IL-10* gene expression (p_LgEV20_ = 0.0379, p_LgEV45_ = 0.0043), whereas 10 µg·mL^−1^ of LgEVs needed 72 h of stimulation to induce *IL-10* upregulation (*p* = 0.0470). 

Taken together, these results indicated that EVs induced MΦ to generate pro- and anti-inflammatory cytokines. Although there were no critical differences between *L. shawi* and *L guyanensis* EVs, *L. shawi* EVs seemed to generate higher cytokines levels (*IL-1β*: *p* = 0.0089, *IL-12p40*: *p* = 0.0008, *TNF-β*: *p* = 0.0165, *IL-4*: *p* < 0.0001, *IL-10*: *p* < 0.0001). Moreover, EV concentration and stimulation time also seemed to play a crucial role in cytokines generation.

### 3.7. L. shawi and L. guyanensis EVs Induce Macrophages to Synthesize NO and Reduce De Novo Urea Production

The microbicide activity of EV-stimulated MΦ was examined by the ability of these cells to metabolize arginine, leading to pro-inflammatory MΦ (M1-MΦ) that produce NO or anti-inflammatory MΦ (M2-MΦ), which leads to urea synthesis. Thereby, M2-MΦ has an important role in tissue repair but favors parasite replication. In contrast, NO is important for the resolution of parasitic infection, as it provides an environment hostile to *Leishmania* survival.

**Figure 9:**
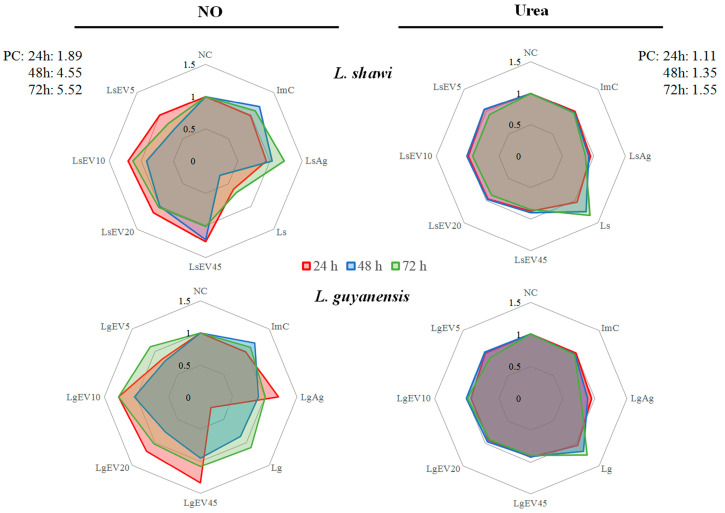
Effect of *L. shawi* and *L. guyanensis* EVs on NO and de novo urea production. MΦ incubated for 24 h, 48 h, and 72 h with 5 (LsEV5; LgEV5), 10 (LsEV10; LgEV10), 20 (LsEV20; LgEV20), and 45 µg·mL^−1^ (LsEV45; LgEV45) of EVs from *L. shawi* and *L. guyanensis*, parasite antigens (LsAg and LgAg), and *L. shawi* (Ls) and *L. guyanensis* (Lg) promastigotes were analyzed by colorimetric assays. Fold change (to resting MΦ) results of three independent assays performed in triplicate are represented by radar graph. NC—negative control (resting MΦ); Positive control (PC -; MΦ incubated with PMA).; ImC—MΦ incubated with negative control of EV isolation method.

MΦ incubated with *L. shawi* and *L. guyanensis* promastigotes for 72 h (*p* = 0.0073) and 48 h (*p* = 0.0228) showed a peak of *de novo* urea production, respectively. On the other hand, MΦ incubated with *L. shawi* EVs (after 72 h p_LsEV5_ = 0.0157, p_LsEV10_ = 0.0392, p_LsEV20_ = 0.0206, p_LsEV45_ = 0.0084), *L. shawi* Ag (p_24h_ = 0.0046, p_48h_ = 0.0044, p_72h_ = 0.0342), *L. guyanensis* EVs (after 72 h p_LgEV20_ = 0.0371, p_LgEV45_ = 0.006), or *L. guyanensis* Ag (p_24h_ = 0.046, p_48h_ = 0.0387, p_72h_ = 0.0002) exhibited a reduction in *de novo* urea production when compared to promastigote infection (Figure 9). 

The production of NO by MΦ exposed to parasites or stimulated with EVs, Ag, and PMA for 24 h, 48 h, and 72 h was analyzed. PMA-stimulated MΦ produced high levels of NO at all time points, indicating that MΦ were functional and able to produce NO. On the contrary, MΦ exposed to promastigotes of both *Leishmania* species showed an early inhibition of NO production (*L. shawi*: p_24h_ *=* 0.0095; *L. guyanensis*: p_24h_ *=* 0.0026). *L. shawi* Ag did not seem to influence NO production, while *L. guyanensis* Ag triggered low NO levels (p_24h_ *=* 0.0006).

MΦ stimulated with 10, 20, and 45 μg·mL^−1^ of EVs shed by both species of *Leishmania* showed an early induction of NO synthesis, followed by a decrease (after 24 h *L.shawi*: p_LsEV10_ = 0.0017, p_LsEV20_ = 0.0016, p_LsEV45_ = 0.0062; *L. guyanensis*: p_LgEV10_ = 0.0028, p_LgEV20_ = 0.0103, p_LgEV45_ = 0.0265). Furthermore, NO synthesis seemed to be dependent on EV concentration, being greater at the highest concentration of EVs.

Taken together, these results indicated that in contrast with the parasite, EVs shed by *L. shawi* and *L. guyanensis* were able to activate MΦ, directing NO production. However, these cutaneous species of *Leishmania* seemed to exert different effects on *de novo* production of urea by MΦ. *L. shawi* EVs impaired urea production, whereas *L. guyanensis* EVs induced an early boost of urea.

### 3.8. L. shawi and L. guyanensis EVs Promote the Expression of MHCI and MHCIMHCII Molecules

MHC molecules complexed with parasite antigens can interact with T lymphocytes by presenting the antigens, which may lead to T cell immune activation. Therefore, it was analyzed the expression of MHC class I and II molecules in MΦ after stimulation with LsEVs and LgEVs to address the potential ability of MΦ to present antigens to lymphocytes. Representative flow cytometry plots and histograms for all experimental conditions are presented in Supplementary Figure S3.

**Figure 10:**
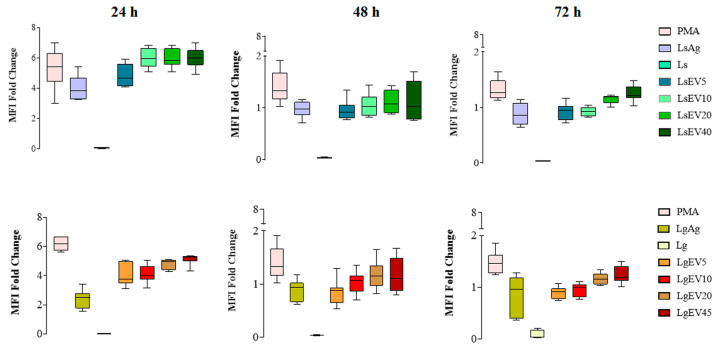
Effect of *L. shawi* and *L. guyanensis* EVs on density of MHCI molecules. MΦ incubated for 24 h, 48 h, and 72 h with 5 (LsEV5; LgEV5), 10 (LsEV10; LgEV10), 20 (LsEV20; LgEV20), and 45 µg·mL^−1^ (LsEV45; LgEV45) of EVs shed by *L. shawi* and *L. guyanensis*, parasite antigens (Ag), and *L. shawi* (Ls) and *L. guyanensis* (Lg) promastigotes were analyzed by multiparametric flow cytometry. Fold changes in MHCI molecule density on MΦ are represented by the MFI (median fluorescence intensity) PMA—MΦ incubated with PMA as a positive control.

**Figure 11:**
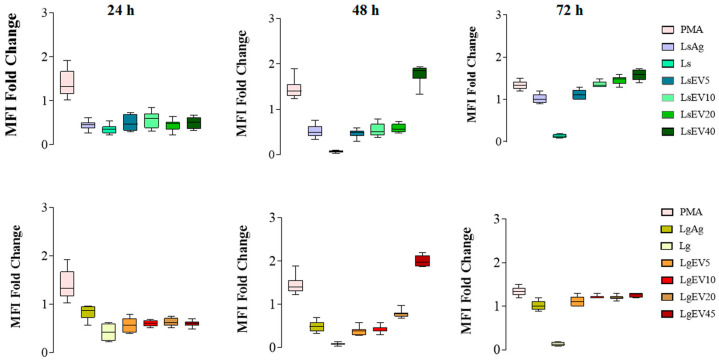
Effect of *L. shawi* and *L. guyanensis* EVs on density of MHCI and MHCII molecules. MΦ incubated for 24 h, 48 h, and 72 h with 5 (LsEV5; LgEV5), 10 (LsEV10; LgEV10), 20 (LsEV20; LgEV20), and 45 µg·mL^−1^ (LsEV45; LgEV45) of EVs shed by *L. shawi* and *L. guyanensis*, parasite antigens (Ag), and *L. shawi* (Ls) and *L. guyanensis* (Lg) promastigotes were analyzed by multiparametric flow cytometry. Fold changes in MHC molecules are represented by the MFI (median fluorescence intensity). PMA—MΦ incubated with PMA as a positive control.

As a positive control of inflammation due to its activation of the nuclear factor-κB, PMA-stimulated MΦ showed a high MFI of MHCI and low MHCII molecules at all time points, indicating that MΦ were able to present antigens. Interestingly, MΦ exposed to *L. shawi* and *L. guyanensis* virulent promastigotes induced an accentuated reduction in the density of MHC molecules (MHCI, MHCII, and MHCIMHCII) that was not recovered during the 72 h of the study (*p* < 0.0001). However, MΦ stimulation with *L. shawi* Ag caused an early increase of the MFI MHCI molecules, followed by a progressive decrease (p_24h_ = 0.0047, p_48h_ = 0.0029). On the other hand, *L.guyanensis* Ag restrained MHCI MFI (p_24h_ = 0.0072, p_48h_ = 0.0099). In contrast, when stimulated by LsEVs and LgEVs, it was observed that the expansion of the MHCI molecules resulted in MFI increasing during the first 24 h of the study (p_LsEV5_ = 0.0024, p_LsEV10_ = 0.0059, p_LsEV20_ = 0.0032, p_LsEV45_ = 0.0013; p_LgEV5_ = 0.0032, p_LgEV10_ = 0.0046, p_LgEV20_ = 0.0093, p_LgEV45_ = 0.0074) (Figure 10). Moreover, EVs had a more discrete effect on the MHCII MFI at all time points (Supplementary Figure S4). After 48 h of stimulation, a reversion was observed in MHCIMHCII MFI (Figure 11) with the highest concentration of LsEVs and LgEVs (45 μg·mL^−1^), driving an accentuated expansion of these MHC molecules (p_LsEV45_ = 0.0018, p_LgEV45_ = 0.0023). At 72 h of stimulation, EVs led to a moderate expansion of these molecules.

Taken together, these results pointed towards the immunological activation of murine MΦ by *L. shawi* and *L. guyanensis* EVs, directing the early increase in MHCI molecules and a later increase in MHCII expression, which could be involved in the antigenic presentation to T cells.

## 4. Discussion

Extracellular vesicles are lipid-bilayer nano-sized vesicles shed by mammal cells and also by parasites, which can circulate in the extracellular microenvironment. Parasite EVs carry macromolecules that can be transferred to host cells, including immune cells, interfering with their normal activity. Despite there being an upsurge of new information about EVs in almost all domains of biomedical sciences in the last few years, the information available on *Leishmania* EVs is still limited, especially in what concerns the influence of EVs on the host’s immune response. Therefore, the current study investigated the modulation of MΦ immune response by EVs shed by two species of *Leishmania* that cause human disease, *L. shawi* and *L. guyanensis*. Both these species belong to the subgenus *Viannia* and, according to Cupolillo and collaborators (1994) [35], are monophyletic species; although few studies are available in the literature, especially on *L. shawi*. *L. shawi* was first described in the Amazon region in 1989 by Lainson and collaborators [36], and in 1991, Shaw and coworkers [37] showed the importance of this species as an agent of CL in the Amazon region and described the pattern of lesions generated, ranging from single to multiple ulcers. However, *L. shawi* usually infects monkeys *Cebus apella* and *Chiropotes satanus*, sloths *Choloepus didactylus* and *Bradypus tridactylus,* as well as coatis *Nasua nasua*, and is rarely detected in humans. *L. guyanensis* constitutes a well-documented agent for MCL in humans and animals and is usually associated with the presence of ulcerative lesions that can progress to mucosal tissue destruction [38]. In these cases, the clinical progression of MCL depends on parasite virulence and the host’s competency of the cell-mediated immune response [39]. Nevertheless, diverse human factors such as deforestation and the spreading of human populations into tropical forest areas for living or tourism, increasing the contact with wild *Leishmania* reservoirs, can lead to an increase in the number of infections for both *Leishmania* species. 

The term EVs describes a heterogeneous population of membrane-enclosed vesicles that cannot replicate and are naturally released by prokaryotic and eukaryotic cells. EVs contain biologically active molecules, including proteins, nucleic acids, lipids, and carbohydrates, and participate in cell-to-cell communication by transferring their cargo content into the recipient cell [40]. The ability of *Leishmania* parasites to secrete EVs was demonstrated in 2010 by Silverman and colleagues [41]. According to the size and biogenesis, EVs from cultured *L. shawi* (LsEVs) and *L. guyanensis* (LgEVs) promastigotes can include exosomes and microvesicles. The small vesicles (30 to 100 nm) of endosomal origin are identified as exosomes. Their biogenesis inside the cell includes the formation of multivesicular bodies by the invagination of endosomal membranes and their release in the extracellular space upon fusion with the plasma membrane [42]. On the other hand, microvesicles are shed directly from the plasma membrane and can have a highly variable size, ranging from 100 to 1000 nm, and their molecular cargo can be specifically enriched [43]. In the present study, LsEVs and LgEVs showed a similar pattern of protein fractions, including a fraction compatible with the presence of active proteases. *Leishmania* virulent promastigotes are coated by a glycocalyx that plays an important role in the initial interaction between the parasite and its host environment. Gp63, also known as major surface protease, leishmanolysin, or promastigote surface protease, is the most abundant protein covering *Leishmania* promastigotes and is considered a major virulence factor in *Leishmania* infection [44,45]. Some of the mechanisms involved in the immune pathogenicity of *Leishmania* infection are a consequence of the ability of gp63 to (i) inactivate the factor C3b of the complement system by generating the C3bi factor that prevents the formation of the membrane attack complex, which leads to promastigotes lysis, (ii) degrade components of the extracellular matrix, facilitating parasite migration, and (iii) cleave intracellular substrates, which ensures intra-macrophage parasite survival and disease progression [46,47]. Interestingly, metalloprotease gp63, a key virulence factor of *Leishmania* parasites, is also a main constituent of *Leishmania* shed EVs, pointing out the potentially crucial role of the vesicles in the early host–parasite communication. [48]. In a recent study, da Silva Lira Filho Alonso and colleagues [49] demonstrated that *L. amazonensis* EVs with different gp63 cargo displayed distinctive macrophage immunomodulatory capabilities and that the high expression of gp63 was essential to sustain the CL pathology, therefore confirming gp63 as a primordial component of EVs in augmenting the cutaneous inflammatory response in *Leishmania* spp. infection. Other proteins can make part of EVs cargo, such as HSP83/90 (heat shock proteins) and *Leishmania* elongation factor 1 α (EF1α). HSP are molecules that play an important role in the immune response, promoting cytokine release by immune cells [50] and acting as chaperones of other molecules, protecting them from degradation caused by the difference of temperature between sand flies (environment temperature 23–26 °C) and the vertebrate host (temperature around 37 °C). EF1α has been considered a parasite virulence factor involved in protein synthesis and downregulation of MΦ microbicide activity by modulating the oxidative pathway [51], contributing to the survival of intracellular parasites in the host. Both HSP70 and EF1α have already been described in previous studies of EVs of *L. infantum*, *L. donovani*, *L. major,* and *L. mexicana* [52,53,54,55]. Although not yet confirmed, HSP and EF1α can make part of *L. shawi* and *L. guyanensis* EVs cargo. Recent studies on *L. donovani* and *L. braziliensis* EVs have described the presence of small non-coding RNAs, particularly tRNA-derived small RNAs in parasitic EVs [56]. However, the potential regulatory effect of these small RNAs was not yet addressed.

Using different analytic methodologies, the present study demonstrated that LsEVs and LgEVs interacted with MΦ, being internalized by the cells. The zeta potential value obtained for LsEVs and LgEVs demonstrated that these nanoparticles were electrically neutral [32], suggesting that *Leishmania* EVs could interact with other cell membranes in a non-disruptive way. Although *L. shawi* and *L. guyanensis* EVs were fast incorporated by MΦ, *L. shawi* EVs seemed to be faster and highly bound to MΦ. Therefore, different LsEVs and LgEVs dynamics may reveal some interesting details of parasite strategy to subvert the host’s immune response, but it can also differ according to the host, reflecting *Leishmania’s* host-specific adaptation. The strong EVs incorporation, which did not interfere with MΦ viability but modulated the gene expression of cytoplasmic (NOD1) and endocytic (TLR9) innate receptors, indicated that EVs (or its cargo) were internalized by MΦ. The incorporation of EVs by MΦ was previously demonstrated by Silverman and collaborators [56], but further studies are needed to clarify the process of incorporation of *Leishmania* EVs by the recipient cells. 

Signalization of PRRs by *Leishmania* parasites triggers a range of intracellular signals that promote the production of immune mediators (cytokines and chemokines), which lead to the activation of the host’s immune system, influencing the type and duration of the immune response. Therefore, the findings of the current study indicated that *L. shawi* and *L. guyanensis* EVs could be recognized by these sensors. In both *L. shawi* and *L. guyanensis* infected MΦ, as well as in EV-stimulated MΦ, the transmembrane innate immune receptor TLR2 exhibited higher upregulation, pointing throughout the recognition of parasite and EVs antigens. This sensor can be activated by parasite lipophosphoglycan, which is highly expressed in the *Leishmania* cell membrane. According to Jafarzadeh and coworkers [57], signalization of TLR2 can have a dual functionality depending on the species of *Leishmania*, triggering a protective immune response or leading to disease development. *L. shawi* and *L. guyanensis* EVs signalization through TLR2 promoted an early boost of NO, which pointed throughout the classical activation of MΦ that it could favor parasite elimination. A study by Polari and coworkers [58] described that in the context of human infection by *L. braziliensis,* the patient’s MΦ increased TLR2 and TLR4 and triggered TNF-α and IL-10. However, despite the fact that EV-stimulated MΦ evidenced TLR2 upregulation during the entire study, NO production was fast abrogated, pointing to a short duration of the activation of MΦ classical pathway. Furthermore, signalization of the TLR4 downstream pathway seemed to be crucial for the efficient expression of inducible nitric oxide synthase (iNOS) [59,60] that is required for NO production. EVs and parasites did not seem to be highly recognized by TLR4, another transmembrane receptor of the MΦ membrane. These findings were in agreement with previous studies reporting that usually, this innate receptor does not seem to be signalized by *Leishmania* antigens [61,62,63,64]. On the other hand, the low levels of the *de novo* urea production indicated that EVs could induce the activation of MΦ alternative pathway. Overall, the engagement of TLRs by parasite antigens appeared to be dependent on the infecting *Leishmania* species and mammal host considered in each study. In addition, diverse *Leishmania* species appeared to trigger different TLRs in order to control the host’s immune response. The endocytic transmembrane TLR9 was signalized by unmethylated CpG motifs of DNA [65], leading to the production of pro-inflammatory cytokines, such as IL-12. *L. shawi* parasites and Ag seemed to be recognized by TLR9, although without generating substantial levels of IL-12. However, *L. shawi* and *L. guyanensis* EVs were early recognized by TLR9 associated with the generation of IL-12p40. Despite the fact that there are only a few available studies characterizing nucleic acids carried out by trypanosomatid EVs, recently, Douanne and colleagues [66] reported gene transfer through *Leishmania* EVs. Thus, it is possible that *L. shawi* and *L. guyanensis* EVs carry parasite DNA that signalizes TLR9 of rodent MΦ.

NOD-like receptor family (NLR) is localized in MΦ cytoplasm and activates signal transduction of the transcription factor NF-κB that induces the expression of pro-inflammatory genes, as is the case of cytokines (e.g., TNF-α, IL-1α, IL-1β, IL-6) and NO production [67,68,69]. Although these cytoplasmatic innate receptors sense intracellular pathogens, limited studies reporting the relation of these sensors with *Leishmania* infection are available. In the current study, NOD1 (NLRC1) seemed to be transiently signalized by *L. shawi* and *L. guyanensis* parasites, as well as by *L. shawi* EVs. In contrast, NOD2 (NLRC2) did not recognize EVs, but promastigotes of both species of *Leishmania* could be signalized through NOD2. The detailed study of PRR profiles appeared as a promising strategy to improve the efficacy of vaccination and therapies with parasite antigens as adjuvants [70]. Together with PRRs engagement, EVs triggered MΦ to generate pro-inflammatory cytokines IL-1β and IL-12, as well as the anti-inflammatory cytokines IL-4 and IL-10. IL-1β and IL-12 had a crucial role in mediating the inflammatory process against *Leishmania* parasites. IL-1β was one of the first cytokines to be produced and promoted the release of other cytokines, including IL-12, a heterodimeric cytokine that modulates the differentiation of Th1 cells. In contrast, cytokines such as IL-4 and IL-10 are responsible for stimulating the differentiation of Th2 cells and regulatory T lymphocytes, respectively, with negative consequences on the activation of MΦ microbicidal pathways. In the current study, IL-4 generation was induced by *L. shawi* and *L. guyanensis* promastigotes and by EVs, being the highest upregulation of this cytokine caused by the parasites. Recently, it was demonstrated that this cytokine could assume a pro-inflammatory role when in the presence of other cytokines, such as TNF-α, promoting parasite control [71,72]. However, in the current study and in contrast with parasites, EVs induced MΦ to generate residual levels of TNF-α. The highest TNF-α upregulation was found in *L. guyanensis* infected MΦ, which could be related to infection pathogenesis since this parasite can cause mucocutaneous leishmaniasis. The highest pathogenicity of *L. guyanensis* could explain the discrete results obtained for LgEVs, as the parasite could more easily escape the host’s immune response and establish the infection. Interestingly, IL-10, a cytokine that regulates inflammatory immune response, was only induced by EVs. Although IL-10 is related to parasite persistence and dissemination and MΦ-M2 polarization, it is also a key cytokine for controlling the exaggeration of the inflammatory response associated with pathology present in parasitic diseases such as malaria, Chagas disease, and leishmaniasis [73,74,75]. Therefore, the generation of IL-10 may be associated with the balancing of the immune response to avoid damage to the host. Overall, the present study illustrated that MΦ polarization is balanced by the combination of TLR2, TLR4, and TLR9, as well as NOD1 and NOD2 activation and different cytokine generation. Moreover, further studies are needed to detail the engagement of other PRRs in the EV signaling pathway. 

**Figure 12:**
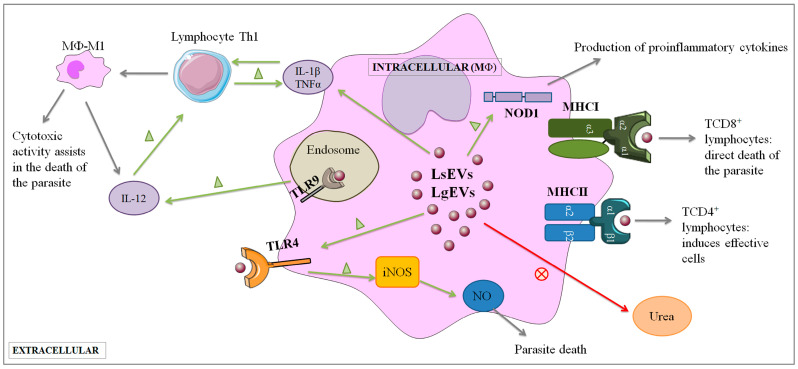
Proposed model for the interplay of *Leishmania* EVs with macrophages. EVs shed by *L. shawi* and *L. guyanensis* can interact with MΦ cellular membrane. EVs appear to interact with innate immune receptors, such as TLR4, NOD1, and TLR9, and can be immunogenic and direct MΦ activity. This includes the generation of pro-inflammatory cytokines (IL-12, IL-1β, and TNF-α) and the expansion of MHCI molecules^,^ inducing the activation of cytotoxic immune response in addition to the production of antimicrobial NO, which can promote parasite inactivation. The Figure was partly designed using Servier Medical Art, provided by Servier, licensed under a Creative Commons Attribution 3.0 unported license (https://creativecommons.org/licenses/by/3.0/).

Despite not mimicking the exact effect of promastigotes in MΦ activation, EVs direct MΦ to generate a mix of regulatory and pro- and anti-inflammatory cytokines, which can lead to a balanced immune response, allowing parasite persistence in the host but avoiding excessive infection that, in the particular case of *L. guyanensis* infection, may prevent the development of mucocutaneous pathology. Furthermore, previous studies demonstrated that the co-inoculation of *Leishmania*-EVs in the host dermis during the phlebotomine blood meal worsened the pathology of the cutaneous lesion with increased expression of inflammatory cytokines [76]. Parasitic EVs can even be involved in drug-resistance mechanisms, as described by Douanne and colleagues [77]. Overall, these data show that *Leishmania* EVs are an essential part of parasite biology and play essential roles in host communication and disease outcomes. The expression of MHC molecules by MΦ is very important as these molecules establish complexes with parasite antigens directing T-cell activation. Virulent *L. shawi* and *L. guyanensis* promastigotes markedly restrain MHCI, and MHCII molecules in MΦ, compromising the capacity of MΦ to present antigens. The decrease in MHCI molecules has been documented as a mechanism of immune subversion in viral infections [78] and cancer [79] but is also observed in *Leishmania* infection. Nyambura and colleagues [80], in a study with *L. donovani,* showed that infected MΦ exhibited a decrease in MHCI and MHCII complex, but CD83 co-stimulatory molecules remained unchanged. The decrease in MHC class I and class II expression on infected cells has also been described in murine studies [81]. Interestingly, LsEVs and LgEVs promote the expansion of MHCI and MHCII expression in MΦ, which indirectly points to the possibility of MΦ presenting the parasite antigens carried out by EVs to CD8^+^ T cells (T cytotoxic lymphocytes) and also to CD4^+^ T cells (T helper lymphocytes). In the context of leishmaniasis, CD8^+^ T cells have been shown to be protective, triggering a cytotoxic immune response that can destroy infected cells, controlling the infection, and preventing disease development [82]. However, increasing evidence indicates that CD8^+^ T cells may also exacerbate disease and the generation of anti-inflammatory cytokines, as well as regulatory cytokines, impairing the development, as a whole, of a predominant protective immune response. Even so, *Leishmania*-EVs appear to be directly involved in the balance of the host’s immune response, either activating cells to exert moderated parasite control or increasing disease severity. The findings of the current study (summarized in Figure 12), although conducted in a murine cell line model, were able to point out that EVs shed by *L. shawi* and *L. guyanensis* carried parasite antigens and also seemed to carry parasite nucleic acids that could be recognized by surface and intracellular PRRs. These EVs are immunogenic and can direct MΦ activity, including the generation of cytokines and the expansion of MHC molecules, which can induce the activation of cytotoxic immune response in addition to the production of antimicrobial NO that can promote parasite inactivation. 

## 5. Conclusions

By delivering parasite macromolecules, *L. shawi* and *L. guyanensis* EVs may play a crucial role in modulating the host’s immune defense, promoting a balanced immune response against the parasite. Thus, since they can be used as vehicles of immune mediators or immunomodulatory drugs, EVs may be a promising target for the development of future prophylactic or therapeutic products/systems for cutaneous leishmaniasis, which is the most common clinical form of leishmaniasis among the disadvantaged human populations. 

## Data Availability

The data presented in this study are available on request from the corresponding author (G.S.-G.). The data are not publicly available due to confidentiality.

## Supplementary Materials

The following supporting information can be downloaded at: https://www.mdpi.com/article/10.3390/cells12081101/s1, Figure S1: Diameter of EVs shed by *L. guyanensis* and *L. shawi* promastigotes; Figure S2: Macrophage incorporation of *L. shawi* and *L. guyanensis* EVs.; Figure S3: Representative histograms of MHCI and MHCII labeled macrophages; Figure S4: Effect of *L. shawi* and *L. guyanensis* EVs on MFI MHCII MΦ; Table S1: Cytokines and PRR primers.
