# Supplementary material for: Insights on Host–Parasite Immunomodulation Mediated by Extracellular Vesicles of Cutaneous Leishmania shawi and Leishmania guyanensis
Source: Cells. 2023 Apr 7;12(8):1101. doi: 10.3390/cells12081101 (PMC10137031; doi:10.3390/cells12081101)
Supplement: Supplementary file 1 [file cells-12-01101-s001.zip › Supplementary Materials Captions.pdf]

Supplementary Figure S1. Diameter of EVs shed by *L. guyanensis* (A) and *L. shawi* (B) promastigotes analyzed by ZetaSizer.

Supplementary Figure S2. Macrophage incorporation of *L. shawi* and *L. guyanensis* EVs. Fluorescence microscope images of MΦ stained with DAPI (blue) and incubated with EVs of *L. shawi* (A) and *L. guyanensis* (B) stained with $DilC_{18}$ (red) (10000x magnification).

Supplementary Figure S3. Representative histograms of MHCI and MHCII labelled macrophages. Median fluorescence intensity (MFI) of macrophages (MØ) infected with *L. shawi* or *L. guyanensis* promastigotes or stimulated with EVs are indicated, as well as the MFI of unstained MØ and staining non-stimulated (resting) MØ. A representative forward scatter (FSC) *vs* side scatter (SSC) dot plot of resting MØ is also included.

Supplementary Figure S4. Effect of *L. shawi* and *L. guyanensis* EVs on MFI MHCII+ MΦ. MΦ incubated for 24 h, 48 h, and 72 h with 5 (LsEV5; LgEV5), 10 (LsEV10; LgEV10), 20 (LsEV20; LgEV20), and 45 µg.mL-1 (LsEV45; LgEV45) of EVs shed by *L. shawi* and *L. guyanensis*, parasite antigens (Ag), and *L. shawi* (Ls) and *L. guyanensis* (Lg) promastigotes were analyzed by multiparametric flow cytometry. Changes in MΦ subset are represented by MFI (median fluorescence intensity) of three independent assays performed in triplicate.

Supplementary Table S1. Cytokines and innate immune pattern recognition receptors primers. Sequences of primer used to evaluate gene expression by RT-PCR are listed as well as the base-pair (bp) of amplificated sequences and the annealing temperature.
