# Supplementary figures and images for: Insights on Host–Parasite Immunomodulation Mediated by Extracellular Vesicles of Cutaneous Leishmania shawi and Leishmania guyanensis
Source: Cells. 2023 Apr 7;12(8):1101. doi: 10.3390/cells12081101 (PMC10137031; doi:10.3390/cells12081101)

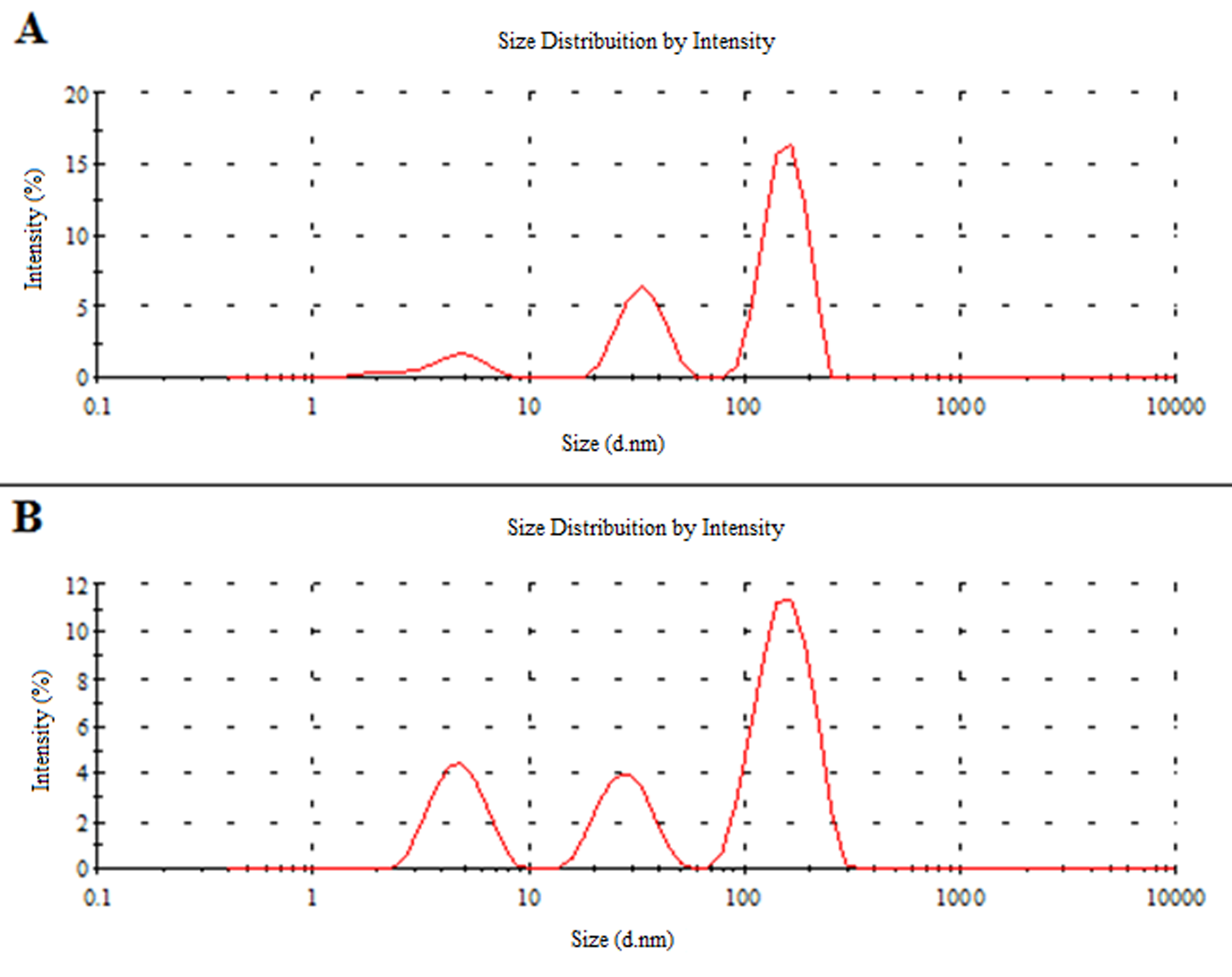

Supplement: Supplementary file 1 [file cells-12-01101-s001.zip › Supplementary Figure S1.tif]

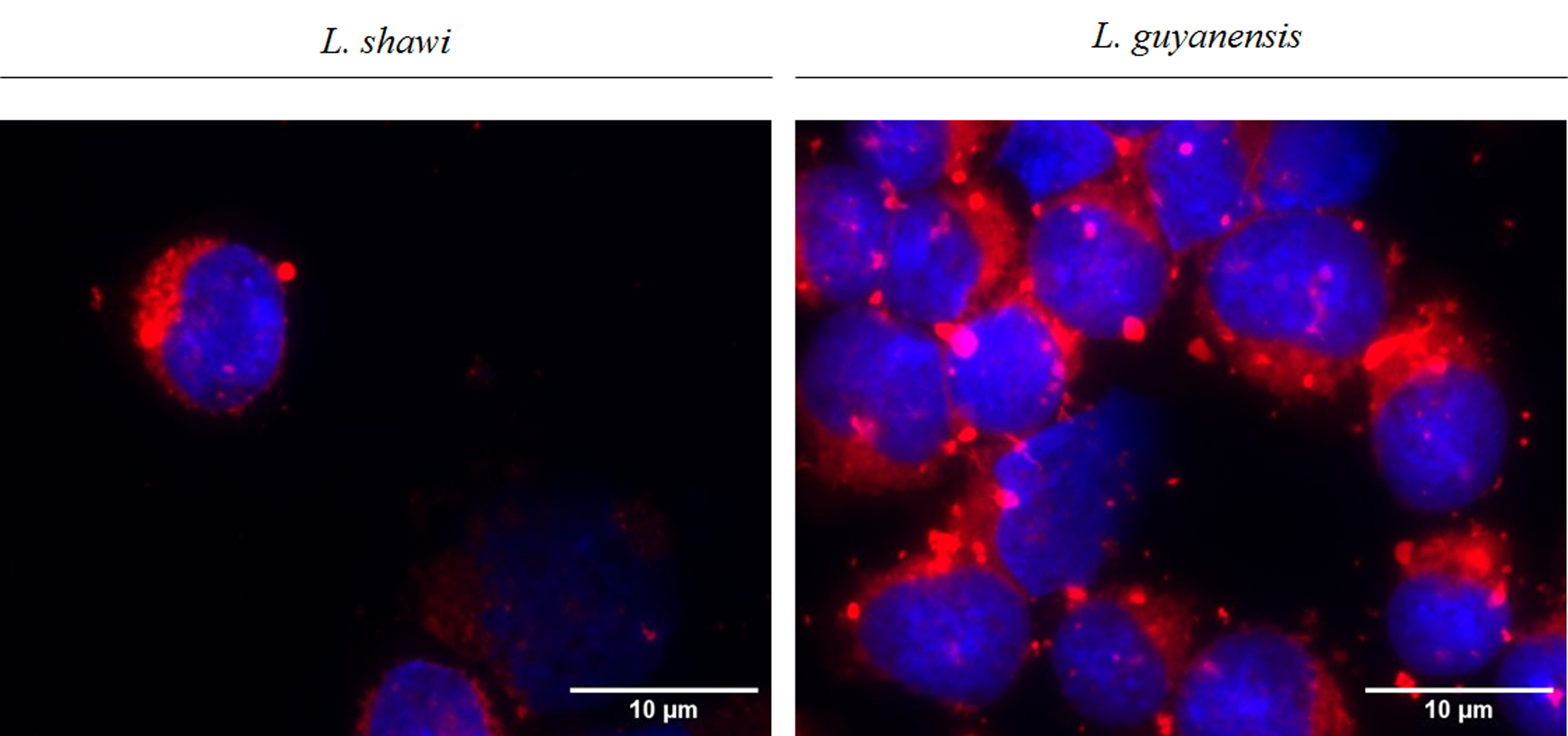

Supplement: Supplementary file 1 [file cells-12-01101-s001.zip › Supplementary Figure S2.tif]

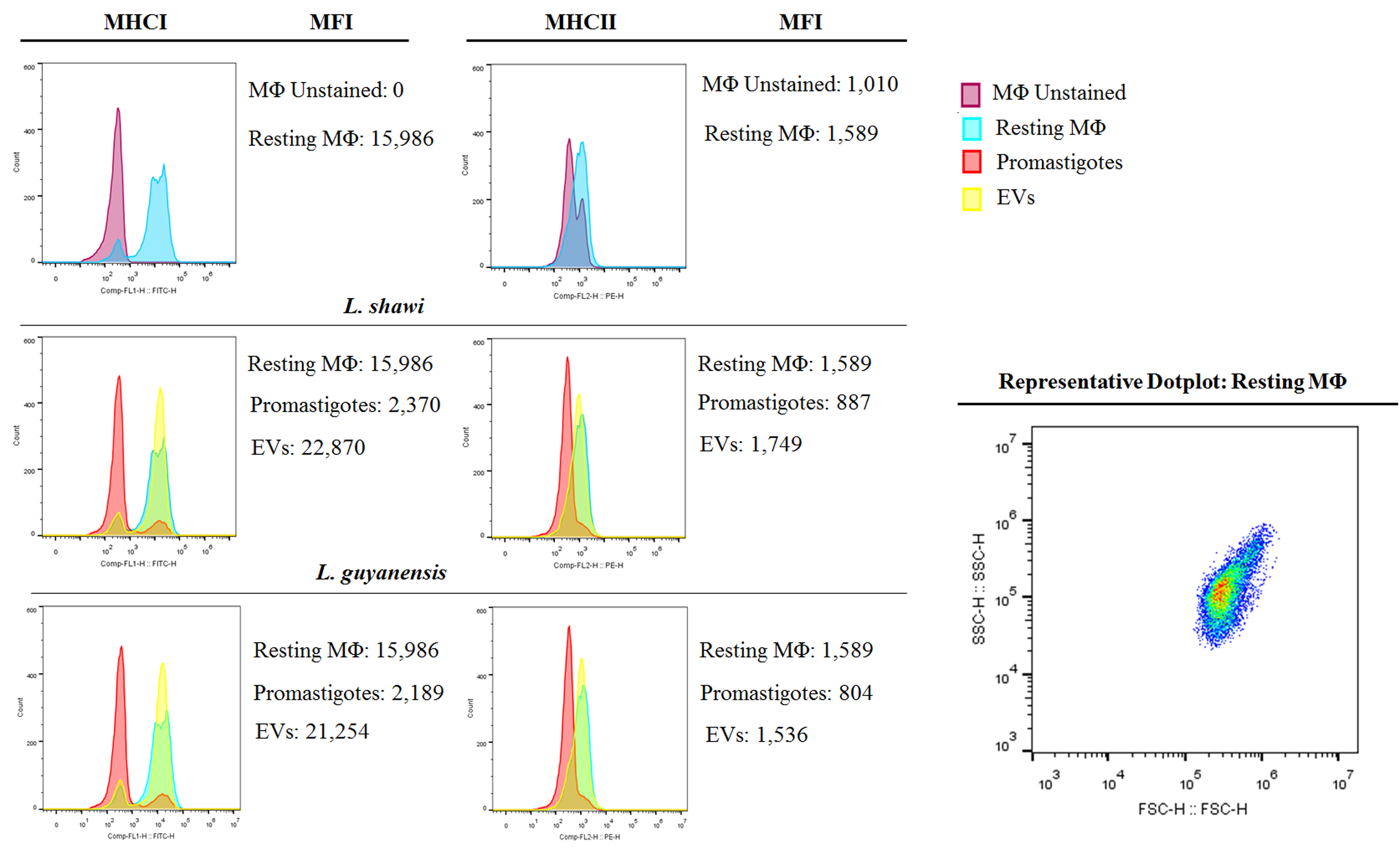

Supplement: Supplementary file 1 [file cells-12-01101-s001.zip › Supplementary Figure S3.tif]

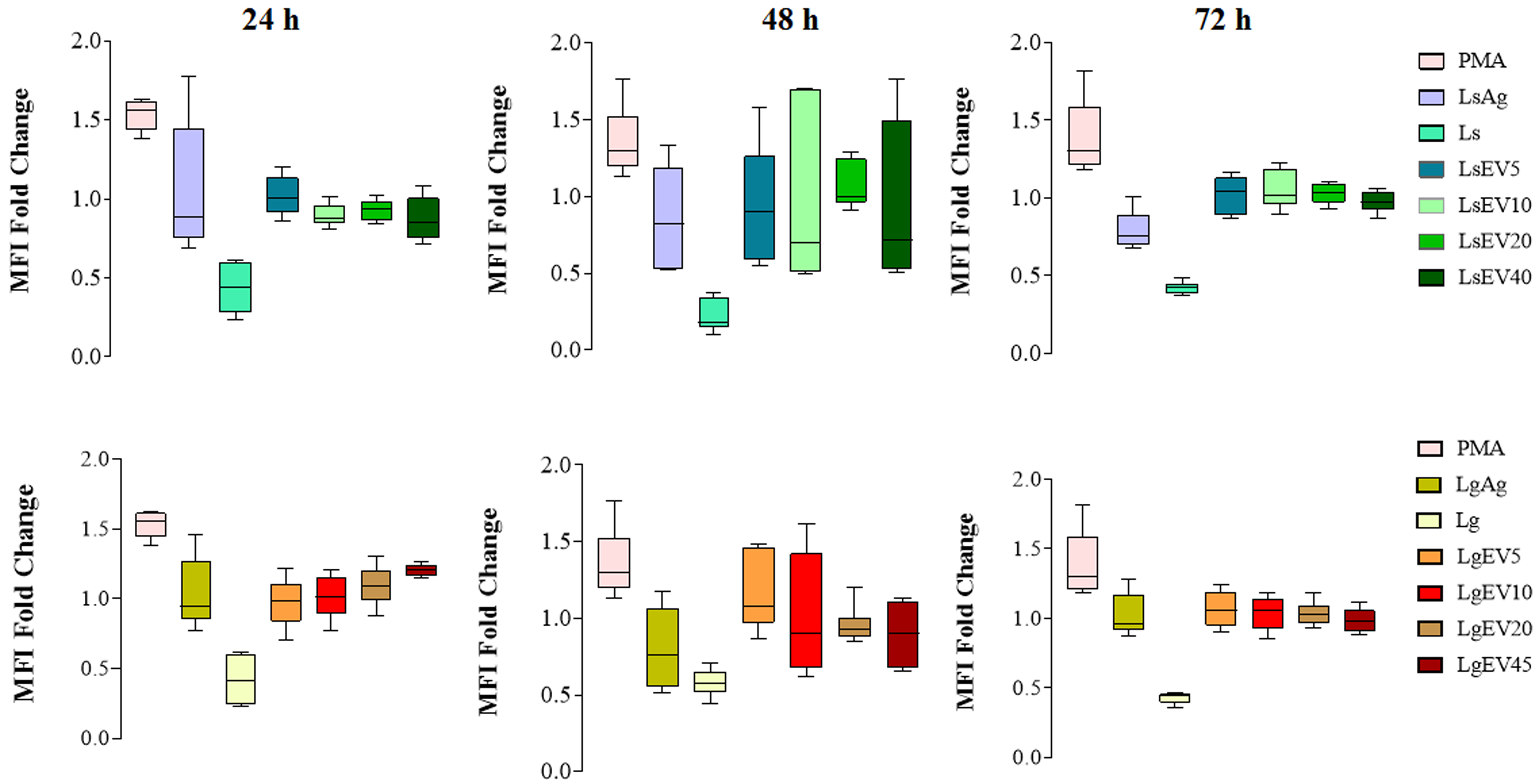

Supplement: Supplementary file 1 [file cells-12-01101-s001.zip › Supplementary Figure S4.tif]

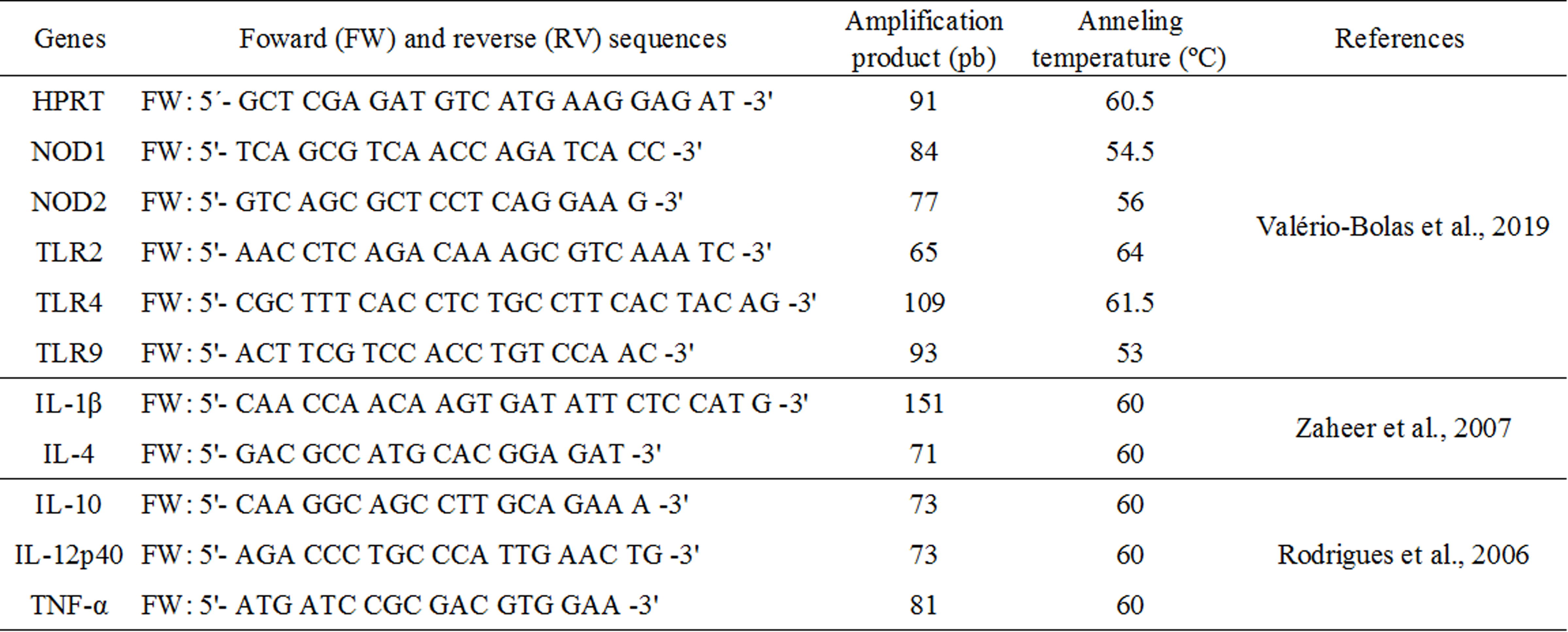

Supplement: Supplementary file 1 [file cells-12-01101-s001.zip › Supplementary Table S1.tif]
